# Development and Pilot Use of the TOMARA Questionnaire for Midwifery-Led Assessment of Ankyloglossia in Newborns

**DOI:** 10.7759/cureus.102325

**Published:** 2026-01-26

**Authors:** Eirini Tomara, Maria Dagla, Evangelia Antoniou, Maria Iliadou, Artemisia Kokkinari, Georgios Iatrakis

**Affiliations:** 1 Department of Midwifery, Research Laboratory of Midwifery Care During Antenatal and Post Natal Period-Breastfeeding, University of West Attica, Athens, GRC; 2 Department of Midwifery, University of West Attica, Athens, GRC

**Keywords:** ankyloglossia, breastfeeding difficulties, breastfeeding support, frenotomy, lingual frenulum, management, tongue mobility restriction, tongue-tied neonates

## Abstract

Background

Ankyloglossia, commonly known as tongue-tie, is diagnosed in neonates when a restrictive lingual frenulum limits tongue mobility and impairs function. This study presents initial data from the newly developed Greek-language questionnaire (Tongue Observation and Mobility Assessment for Oral Restrictions due to Ankyloglossia (TOMARA)), used in conjunction with the Assessment Tool for Lingual Frenulum Function (ATLFF). The objective was to document clinical examination findings of the infant oral cavity and the breastfeeding challenges experienced by each mother-infant dyad under midwifery-led management.

Methodology

The psychometric properties of the TOMARA questionnaire were evaluated by assessing internal consistency, construct validity, and predictive validity. Of the 347 neonates examined, 51 showed clinical indications of ankyloglossia; the diagnosis was confirmed in 48 cases, and 44 subsequently underwent frenotomy. The breastfeeding difficulties of the dyad were recorded before and after the intervention.

Results

The most frequently observed breastfeeding difficulties included poor latch (82.4%) and prolonged feeding duration (82.4%). Mothers frequently reported persistent pain during breastfeeding (84.3%), along with irritated (94.1%) and/or cracked nipples (39.2%). Functional limitations of the lingual frenulum were significantly associated with both maternal (p = 0.041) and neonatal symptoms (p = 0.004). In treated cases, most symptoms significantly improved or resolved. This was reflected in breastfeeding duration, with 86.4% of mothers continuing to breastfeed for more than six months, in alignment with World Health Organization recommendations.

Conclusions

Despite the relatively small size of our study, the findings suggest that effective management of ankyloglossia relies on the careful selection and thorough evaluation of tongue-tied infants. The functional performance of the lingual frenulum is associated with fewer breastfeeding difficulties for both mother and infant.

## Introduction

The lingual frenulum is a dynamic fold that connects the tongue to the floor of the mouth and displays considerable anatomical variation among neonates. The characteristics of the frenulum may have a strong impact on tongue mobility, and when this mobility is restricted, the condition is termed ankyloglossia [1,2]. Given the critical role of tongue mobility in the breastfeeding process, many cases of ankyloglossia are often identified due to difficulties encountered during breastfeeding [3]. According to the World Health Organization (WHO), breastfeeding should ideally begin within the first two hours after birth, be exclusive for the first six months of life, and continue alongside the introduction of solid foods for at least the first two years [4]. Human breast milk is a biologically adaptive fluid, specifically produced by the mother to meet the nutritional and developmental needs of her infant, thereby supporting optimal growth and development [5,6].

Midwives, as healthcare professionals who provide personalized breastfeeding counseling, are frequently called upon to identify and manage the implications of ankyloglossia. Although recent years have seen an increase in publications and heightened awareness regarding this condition, numerous issues remain unresolved or insufficiently defined [3]. A review of the literature reveals contributions from pediatricians, otolaryngologists, plastic surgeons, speech therapists, midwives, and lactation consultants, each bringing a unique professional perspective [2]. Consequently, each healthcare professional, depending on their area of expertise, emphasizes different aspects in identifying neonatal ankyloglossia, either focusing on gross anatomical features or on a combination of anatomical and functional clinical findings. Anatomical or morphological features refer to the shape of the tongue, the point of attachment of the lingual frenulum to both the tongue and the floor of the mouth, as well as the thickness and elasticity of the frenulum. Functional features include tongue extension, elevation, and lateralization. This diversity of professional involvement and the lack of consensus on fundamental aspects are the main reasons for the ongoing controversies within the field of ankyloglossia research. Two of the most important questions raised are which newborns will ultimately be affected, and in what way.

A comprehensive clinical examination of the newborn’s oral cavity, detailed maternal history concerning breastfeeding, and direct observation of a breastfeeding session by a healthcare professional are essential steps in identifying ankyloglossia and its symptoms [2,7,8]. The findings from inspection of the oral cavity and palpation of the frenulum may reveal morphological or functional impairments related to tongue mobility [7,9]. Restrictions in tongue mobility can lead to breastfeeding difficulties that may result in early weaning [10]. Distinguishing between normal and pathological ranges of tongue motion and understanding the anatomy of the oral cavity are vital.

This study aimed to document clinical examination findings of the infant oral cavity and the breastfeeding challenges experienced by each mother-infant dyad under midwifery-led management. The midwifery-led approach in this study focused on the initial conservative management of the challenges faced by the mother, the newborn, or both, along with personalized counseling. In cases where tongue functionality appeared compromised and symptoms persisted despite conservative measures, infants were referred for pediatric surgical evaluation. Parents retained the right to choose the pediatric surgeon responsible for reassessing the frenulum, establishing a definitive diagnosis, and performing surgical intervention if indicated. While midwives are not authorized to provide a medical diagnosis, they are trained to recognize clinical indicators that warrant timely referral and interdisciplinary collaboration. The ultimate goal is to ensure that breastfeeding is both effective and satisfying for the mother and infant, thereby promoting a positive breastfeeding experience and reinforcing the maternal-infant bond.

To our knowledge, this is one of the first prospective studies in Greece to explore neonatal ankyloglossia using both a validated clinical assessment tool (Assessment Tool for Lingual Frenulum Function (ATLFF)) and a newly developed observational questionnaire presented in Greek (Tongue Observation and Mobility Assessment for Oral Restrictions due to Ankyloglossia (TOMARA)). In addition to evaluating breastfeeding outcomes, this study contributes to the psychometric validation of a new questionnaire designed for routine use in midwifery-led care. The findings aim to inform clinical decision-making and highlight the midwife’s role in early detection and management of ankyloglossia.

Part of the data included in this article was previously presented as a poster at the 8th EMA (European Midwives Associations) Educational Conference, held on October 24-25, 2025.

## Materials and methods

The initial number of infants examined within the context of the study was 347. Of these, 51 infants were identified with characteristics indicative of restricted tongue mobility and thus comprised the study’s case group. Due to the severe breastfeeding difficulties experienced by these mother-infant dyads, establishing a control group was deemed ethically challenging. The study protocol was initiated in 2019, and between 2019 and 2021, the research team developed the TOMARA questionnaire. The administration of the questionnaire and the data collection process began in mid-March 2021 and continued until February 2025. All newborns included in the study were born at the “Rea” Maternity and Gynecology Clinic in Athens, Greece.

The inclusion criteria were mothers of Greek origin who delivered full-term infants (≥37 weeks of gestation) with a birth weight greater than 2,500 g, either through vaginal delivery or cesarean section. Supportive midwifery care was provided from the immediate postpartum period, beginning with the first breastfeeding session and sustained through daily visits during the first four to five days postpartum. During these visits, in addition to monitoring the breastfeeding process and providing individualized counseling, the midwife conducted a thorough examination of the newborns’ oral cavity with the aim of early identification of signs suggestive of restricted tongue mobility. Exclusion criteria included infants born before the 37th week of gestation, full-term infants requiring hospitalization in a neonatal intensive care unit (NICU), neonates diagnosed with perinatal illnesses, severe craniofacial anomalies, or genetic syndromes, mothers under the age of 18 years, and mothers who chose to cease breastfeeding immediately postpartum.

For each mother-infant dyad, detailed documentation was undertaken regarding breastfeeding difficulties, and initial interventions were implemented using conservative measures alongside individualized counseling to improve the breastfeeding process. In cases where difficulties persisted, the dyads were referred to a pediatric surgeon for further evaluation. The researchers were not aware of which pediatric surgeon the parents would choose, nor of the surgeon’s final decision regarding whether to perform a frenotomy. If a diagnosis of ankyloglossia was confirmed and informed parental consent was obtained, therapeutic intervention was performed through frenotomy. Following the surgical procedure, a follow-up consultation was conducted with the parents to reassess breastfeeding progress. Within one to two weeks post-procedure, mothers were contacted by phone to complete a structured symptom-tracking questionnaire for the mother-infant dyad. Additionally, to ensure comprehensive documentation of the entire breastfeeding course, a follow-up telephone contact was conducted at a later stage to record the duration of exclusive breastfeeding and/or combined feeding with formula, continuing until weaning (natural weaning, parent-led weaning, or medically indicated weaning) had occurred.

The observations from the oral examination of the neonates were conducted using the newly developed TOMARA questionnaire, in conjunction with the ATLFF by Dr. Hazelbaker [11]. The new form, titled Tongue Observation and Mobility Assessment for Oral Restrictions due to Ankyloglossia, includes seven illustrated questions related to the lingual frenulum and three related to the upper lip frenulum, all presented in Greek (Appendix 1-4). Additionally, it gathers relevant information from the neonate’s medical history and breastfeeding experience. To conduct the study, the psychometric properties of the TOMARA questionnaire were evaluated by examining its internal consistency (using Cronbach’s alpha coefficient), construct validity (through factor analysis), and predictive validity (by correlating its results with those of the ATLFF).

To document the difficulties experienced by mothers and their newborns or infants during breastfeeding, two distinct evaluation forms were developed (Appendix 5). The first was completed before referral to a pediatric surgeon and before a definitive diagnosis was established, while the second was completed following the frenotomy procedure. Regarding neonatal symptoms, the following issues were recorded: poor weight gain and growth, the need for supplementary feeding with modified cow’s milk formula, difficulty latching onto the breast, prolonged breastfeeding duration, and the audible “clicking” sound during feeding. For mothers, recorded difficulties included intense pain during breastfeeding, irritated, cracked, or bleeding nipples, plugged ducts, frequent breast engorgement, and mastitis. At the conclusion of the first evaluation form, administered before frenotomy, the midwife documented her clinical decision regarding referral to a pediatric surgeon. Additionally, the parents’ intent and the primary reason that led them to seek surgical consultation were recorded.

The post-intervention form, completed after the frenotomy procedure, documented the evolution of symptoms within the mother-infant dyad (Appendix 6, 7). For each previously identified symptom, the recorded outcome was categorized as complete resolution, partial improvement, or no improvement following the therapeutic intervention. This form also included detailed procedural information: the date of the frenotomy, the method used, the final diagnosis and classification of tongue-tie, and any complications that occurred. Furthermore, the total duration of breastfeeding for each dyad was recorded. In cases where breastfeeding had not yet concluded by the end of the data collection period, the number of months of breastfeeding up to February 2025 was documented.

To ensure compliance with ethical and deontological standards, a confidentiality agreement and informed consent form were completed for each case included in the study. These documents secured parental consent for the participation of both the parent and the newborn in the research process. The primary objective was to protect personal data and to ensure the anonymity of participants throughout the study’s implementation and the subsequent publication of its findings.

Data analysis

Initial data recording and coding were performed using Microsoft Excel. Subsequently, a database was created in SPSS Statistics version 22 (IBM Corp., Armonk, NY, USA) to conduct the statistical analysis. Descriptive statistics were applied to summarize the characteristics of the study sample (51 mothers and newborns/infants). Specifically, quantitative variables were described using measures of central tendency and dispersion (mean, median, standard deviation, minimum, and maximum), while qualitative variables were presented using frequency distributions (n, %).

The psychometric properties of the TOMARA questionnaire were evaluated by assessing internal consistency, construct validity, and predictive validity. Internal consistency was examined using Cronbach’s alpha coefficient. Construct validity was assessed through exploratory factor analysis (EFA) to investigate the underlying factor structure of the questionnaire. Normality of data distributions was evaluated using the Kolmogorov-Smirnov and Shapiro-Wilk tests, and descriptive indices including means, standard deviations, skewness, and kurtosis were calculated for the individual scales. Predictive validity was assessed by examining the correlation between TOMARA questionnaire scores and those of ATLFF using Spearman’s correlation coefficient. Statistical significance was set at p-values <0.05.

## Results

Psychometric validation of the ATLFF tool and the TOMARA questionnaire in our study

Initially, EFA was conducted to examine the factor structures as they emerged within our sample. According to MacCallum et al., when communalities are high (>0.6) and factor loadings are strong (>0.6), sample sizes smaller than 100 can be considered adequate for EFA [12]. In the present study, although the sample consisted of only 51 participants, thus supporting the adequacy of the smaller sample size for exploratory purposes on this criterion. Factors extracted from both scales were those with eigenvalues greater than 1.00, following the Kaiser-Guttman criterion, which is considered a primary rule for determining the number of factors to retain. Furthermore, as suggested in the literature, an item is considered to load on a factor if its loading exceeds 0.40 and it does not display significant secondary loadings on other factors [13,14].

According to the Kaiser-Meyer-Olkin (KMO) measure (KMO = 0.79) for the ATLFF scale, the sample was deemed adequate for factor analysis. The correlation matrix of the 12 items was also appropriate based on Bartlett’s test of sphericity (χ² = 246.69, df = 66, p < 0.001). Two factors were extracted, accounting for 53.32% of the total variance. The first factor, comprising the functional characteristics, explained 28.68% of the variance (8 items), while the second factor, consisting of the morphological characteristics, accounted for 24.65% of the variance (4 items) (Table 1).

**Table 1:** Psychometric validation of the ATLFF tool and the TOMARA questionnaire. ATLFF = Assessment Tool for Lingual Frenulum Function; TOMARA = Tongue Observation and Mobility Assessment for Oral Restrictions due to Ankyloglossia; EFA = exploratory factor analysis; PCA = principal component analysis

Psychometric property	Method/Statistical test	ATLFF	TOMARA questionnaire
Sample adequacy	Kaiser-Meyer-Olkin	0.79	0.72
Bartlett’s test of sphericity	χ²	246.69	167.68
	df	66	36
	p-value	<0.001	<0.001
Factor extraction	EFA/PCA (eigenvalues >1)	2	2
Total variance explained	Percentage of variance (%)	53.32	58.11
Factor 1	Description	Functional characteristics	Lingual frenulum
	Number of items	8	6
	Variance explained (%)	28.68	35.61
Factor 2	Description	Morphological characteristics	Lip frenulum
	Number of items	4	3
	Variance explained (%)	24.65	22.50
Internal consistency	Cronbach’s alpha	Functional: 0.80	Lingual: 0.81
		Morphological: 0.44	Labial: 0.72
		Morphological (without elasticity feature): 0.72	-

An exploratory principal component analysis (PCA) was conducted with orthogonal rotation (varimax with Kaiser normalization). The sample adequacy was confirmed by the KMO measure (KMO = 0.72), and the correlation matrix was deemed suitable for factor analysis according to Bartlett’s test of sphericity (χ² = 167.68, df = 36, p < 0.001). Two factors emerged from the analysis, explaining a total of 58.11% of the variance. The first factor, associated with the lingual frenulum, accounted for 35.61% of the variance and included six items. The second factor, associated with the lip frenulum, explained 22.50% of the variance and included three items. No cross-loadings were observed, indicating a clear factor structure. Cronbach’s alpha was calculated at 0.81 for the lingual frenulum subscale and 0.72 for the labial frenulum subscale, indicating strong internal consistency. To further establish the predictive validity, its scores were compared to the ATLFF tool, specifically its morphological and functional components related to the frenulum. Spearman’s rho correlations were strong and statistically significant (ρ = 0.74 and ρ = 0.75), demonstrating the TOMARA questionnaire’s excellent criterion-related validity in this sample.

Characteristics of the sample

The majority of ankyloglossia cases were boys, accounting for 60.8% (n = 31) of the sample, whereas girls comprised 39.2% (n = 20). Regarding birth weight, the most common category was 3,000-3,499 g (52.9%, n = 27). Most neonates were born between 39 and 39+6 weeks of gestation (33.3%, n = 17), with the majority delivered via vaginal birth (68.6%, n = 35). A notable proportion of mothers had previous breastfeeding experience (35.29%, n = 18), although the majority had not breastfed in the past (64.71%, n = 33) (Table 2). Correlation between the number of neonatal feeding difficulties and the morphological and functional scores based on the ATLFF assessment tool.

**Table 2:** Distribution of absolute and relative frequencies by gender, birth weight, gestational age, type of delivery, previous breastfeeding experience, and its duration.

Characteristics of the sample	N	%
Gender		
Boys	31	60.8%
Girls	20	39.2%
Total	51	100%
Birth weight (in g)		
2,500–2,999	9	17.6%
3,000–3,499	27	52.9%
3,500–4,000	13	25.5%
>4,000	2	3.9%
Total	51	100%
Weeks of gestation		
37–37^+6^	8	15.7%
38–38^+6^	8	15.7%
39–39^+6^	17	33.3%
40–40^+6^	16	31.4%
41–42	2	3.9%
Total	51	100%
Type of delivery		
Vaginal delivery	35	68.6%
Cesarean section	16	31.4%
Total	51	100%
Previous breastfeeding experience		
Yes	18	35.29%
No	33	64.71%
Total	51	100%
Duration of breastfeeding experience (in months)		
<6	4	22.2%
6–12	8	44.4%
>12	6	33.4%
Total	18	100%

Clinical examination of the oral cavity

The most commonly observed shape was rounded, recorded in 70.6% (n = 36) of the sample. This was followed by a V-shaped tongue with a central notch (21.6%, n = 11), while the heart-shaped tongue was identified in a smaller proportion of cases (7.8%, n = 4). Concerning the attachment of the frenulum to the tongue, the most frequent site was from the middle of the tongue and posteriorly (51.0%, n = 26). Anterior attachment, including at the tip of the tongue, was documented in 33.3% (n = 17), while the least common point of attachment was at the mid-tongue region (15.7%, n = 8).

Regarding the attachment of the lingual frenulum to the floor of the mouth, in most cases, it was connected to the mucosal layer (64.7%, n = 33), while in a smaller percentage, the attachment was observed either on the inner lower surface of the mandible or on the alveolar ridge (35.3%, n = 18). In terms of the elasticity and thickness of the frenulum, it was most commonly characterized as mildly elastic (52.9%, n = 27). In 21.6% (n = 11) of cases, the frenulum was highly elastic and thin, whereas in 25.5% (n = 13) of neonates, it was described as non-elastic and thick. Concerning tongue mobility, the majority of neonates exhibited non-wavelike or uncoordinated movements (64.7%, n = 33), while wavelike movement was observed in 31.4% (n = 16) of cases. A limited number of neonates showed very mild or nearly absent tongue movement (3.9%, n = 2).

Based on the degree of anterior tongue protrusion, the most common position observed was tethered behind the alveolar ridge (56.9%, n = 29). In 35.3% (n = 18) of cases, the tongue reached the border of the lips, while in a small percentage (7.8%, n = 4), it was able to extend fully beyond the lips. In terms of tongue position during neonatal crying, the most frequently observed posture was elevation of the lateral edges or both the tip and sides of the tongue along the midline, resulting in the characteristic “spoon-shaped” appearance (66.7%, n = 34). Fewer neonates exhibited the tongue resting at the floor of the oral cavity, with or without lateral elevation (19.6%, n = 10), and the least common observation was a raised tongue near the palate or elevated along the midline (13.7%, n = 7) (Table 3).

**Table 3:** Clinical examination findings of the oral cavity.

Oral cavity characteristic	Feature	Anatomical findings	n (%)
Tongue shape	Shape	Rounded	36 (70.6%)
		V-shaped with a central notch	11 (21.6%)
		Heart-shaped	4 (7.8%)
Lingual frenulum attachment to the tongue	Attachment site	Middle/Posterior tongue	26 (51.0%)
		Anterior (including tip)	17 (33.3%)
		Mid-tongue region	8 (15.7%)
Lingual frenulum attachment to the floor of the mouth	Attachment site	Mucosal layer	33 (64.7%)
		Mandible/Alveolar ridge	18 (35.3%)
Lingual frenulum elasticity and thickness	Elasticity	Mildly elastic	27 (52.9%)
		Highly elastic and thin	11 (21.6%)
		Thick and non-elastic	13 (25.5%)
Tongue mobility	Movement pattern	Non-wavelike/Uncoordinated	33 (64.7%)
		Wavelike	16 (31.4%)
		Very mild or nearly absent	2 (3.9%)
Anterior tongue protrusion	Protrusion ability	Tethered behind the alveolar ridge	29 (56.9%)
		Reaches lip border	18 (35.3%)
		Extends beyond lips	4 (7.8%)
Tongue position during crying	Tongue posture	Spoon-shaped elevation	34 (66.7%)
		Resting on the floor of the mouth	10 (19.6%)
		Raised near the palate/midline	7 (13.7%)
Upper lip frenulum elevation	Elevation	Restricted	39 (76.5%)
		Not restricted	12 (23.5%)
Upper lip frenulum elasticity	Elasticity	Mildly elastic	42 (82.4%)
		Highly elastic and thin	5 (9.8%)
		Thick and non-elastic	4 (7.8%)
Upper lip frenulum attachment to the maxilla	Attachment site	Alveolar ridge	34 (66.7%)
		Upper/Middle maxilla	13 (25.5%)
		Near the hard palate	4 (7.8%)

Examination of the upper lip frenulum revealed restricted elevation in the majority of cases, specifically in 76.5% (n = 39) of the newborns, whereas no such restrictions were observed in 23.5% (n = 12). Concerning the characteristics of the upper lip frenulum, the majority of cases (82.4%, n = 42) were described as mildly elastic. A highly elastic and thin frenulum was observed in 9.8% (n = 5), whereas a thick and non-elastic frenulum was identified in 7.8% (n = 4) of neonates. The most frequent attachment site of the upper lip frenulum to the maxilla was at the alveolar ridge (66.7%, n = 34). This was followed by attachment to the upper or middle section of the maxilla (25.5%, n = 13), while attachment near the hard palate was the least common finding (7.8%, n = 4).

The analysis of the lingual frenulum and tongue characteristics using the ATLFF tool simultaneously revealed that the vast majority of the newborns, 48 out of 51 (94.1%), were classified in the third category, as defined by the tool’s creator. This category refers to cases where tongue mobility is compromised, and a frenotomy is recommended. In contrast, three cases demonstrated normal tongue function and therefore did not require any therapeutic intervention (Table 4).

**Table 4:** Classification of the sample cases according to the final scores obtained from the completion of the ATLFF tool. ATLFF = Assessment Tool for Lingual Frenulum Function

Final score	n	%
14 = Perfect function score regardless of the appearance item score. Surgical treatment is not recommended	-	-
11 = Acceptable function score only if the appearance item score is ≥8	3	5.9%
<11 = Function score indicates function impaired. Frenotomy should be considered if management fails. Frenotomy is necessary if the appearance is <8	48	94.1%
Total	51	100%

Breastfeeding symptoms of the mother-infant dyad before the surgical interventions

Taking into consideration the difficulties experienced by newborns during breastfeeding, it was found that the majority (82.4%, n = 42) had difficulty latching onto the nipple. Additionally, in 56.9% (n = 29) of the cases, the characteristic clicking sound of the tongue was recorded during breastfeeding. Another common feature among newborns with restricted tongue function was prolonged breastfeeding duration (82.4%, n = 42), which may reflect difficulty in obtaining an adequate amount of milk. At the same time, a significant proportion (37.3%, n = 19) required supplementary feeding with formula. When examining symptoms associated with growth and feeding, only a minority of newborns presented with notable challenges. Specifically, inadequate weight gain was recorded in 19.6% (n = 10) of cases, delayed recovery of birth weight in 17.6% (n = 9), and weight loss in 15.7% (n = 8) (Table 5).

**Table 5:** Absolute and relative frequencies of the symptoms concerning the newborn before the surgical intervention. ^1^: Less than 20 g per day. ^2^: Difficulty in regaining birth weight within 14 days after birth. ^3^: More than 10% of the birthweight. ^4^: Prolonged feeding time is defined as more than half an hour in each breast.

Neonatal symptoms		n	%
Ιnadequate weight gain^1^	No	41	80.4%
	Yes	10	19.6%
	Total	51	100%
Difficulty regaining birth weight^2^	No	42	82.4%
	Yes	9	17.6%
	Total	51	100%
Weight loss^3^	No	43	84.3%
	Yes	8	15.7%
	Total	51	100%
Difficulty latching onto the nipple	No	9	17.6%
	Yes	42	82.4%
	Total	51	100%
Clicking sound during breastfeeding	No	22	43.1%
	Yes	29	56.9%
	Total	51	100%
Prolonged breastfeeding sessions^4^	No	9	17.6%
	Yes	42	82.4%
	Total	51	100%
Supplemental feeding with infant formula	No	32	62.7%
	Yes	19	37.3%
	Total	51	100%

In addition to the symptoms exhibited by newborns during breastfeeding, symptoms reported by mothers were also recorded (Table 6). The most dominant symptom was pain (84.3%): nearly half (49.0%, n = 25) of the mothers reported mild pain (intensity ≤5 on the pain scale) throughout the breastfeeding process, while a significant proportion (35.3%, n = 18) experienced severe pain (intensity >5). There was also a high prevalence of nipple irritation, with 94.1% (n = 48) of mothers experiencing this symptom, and only a small percentage (5.9%, n = 3) reporting no irritation. Beyond irritation, specific difficulties such as cracked or bleeding nipples were recorded in 39.2% (n = 20) of the mothers. Additional symptoms reported by mothers included blocked milk ducts in 7.8% (n = 4), breast engorgement in 13.7% (n = 7), and mastitis in 7.8% (n = 4) of cases.

**Table 6:** Absolute and relative frequencies of the maternal symptoms before the surgical intervention. ^1^: Intensity >5 on the pain scale.^2^: Intensity ≤5 on the pain scale.

Maternal symptoms		n	%
Severe pain during breastfeeding^1^	No	33	64.7%
	Yes	18	35.3%
	Total	51	100%
Mild pain during breastfeeding^2^	No	26	51.0%
	Yes	25	49.0%
	Total	51	100%
Nipple irritation	No	3	5.9%
	Yes	48	94.1%
	Total	51	100%
Cracked or bleeding nipples	No	31	60.8%
	Yes	20	39.2%
	Total	51	100%
Blocked milk ducts	No	47	92.2%
	Yes	4	7.8%
	Total	51	100%
Breast engorgement	No	44	86.3%
	Yes	7	13.7%
	Total	51	100%
Mastitis	No	47	92.2%
	Yes	4	7.8%
	Total	51	100%

Referral of cases, parents’ decision, and surgical intervention

Taking into consideration the anatomical and morphological characteristics, as well as the difficulties presented by the dyad during breastfeeding, 48 out of the total 51 cases were referred to a pediatric surgeon due to strong clinical indications (Table 7). Of the 48 initially referred cases, 44 eventually visited a pediatric surgeon. The parents of the remaining four declined the referral. The parents of the 44 newborns who were ultimately referred were asked to indicate the main reason they agreed to meet with the pediatric surgeon. Their responses are presented in Table 7, with the three most frequent ones being: to improve the breastfeeding process, to relieve breastfeeding-related pain, and to prevent future complications.

**Table 7:** Absolute and relative frequencies regarding the referral of cases and the parents’ decision.

Referral and follow-up summary	n	%
Referral status		
Yes	48	94.1%
No	3	5.9%
Total	51	100.0%
Parental decision regarding pediatric surgeon visit		
Positive	44	86.3%
Negative	4	7.8%
Follow-up	3	5.9%
Total	51	100%
Main reason for visiting the pediatric surgeon		
Pain relief during breastfeeding	14	31.8%
Improvement of the breastfeeding process	15	34.2%
Prevention of future complications	13	29.5%
Infant’s excessive distress	2	4.5%
Total	44	100%

Out of the 44 cases that were referred, the diagnosis of ankyloglossia was confirmed in all, while in 38 of them, a restrictive upper lip frenulum was also present. Regarding the number of days that elapsed until the frenotomy (from birth), the most common time frame was the first week of life (1-7 days), accounting for 36.4% (n = 16). This was followed by the periods of 8-14 days (20.5%, n = 9), 22-30 days (22.7%, n = 10), and 15-21 days (15.9%, n = 7), while in a few cases the procedure was performed after 30 days (4.5%, n = 2) (Table 8). Concerning the method of frenotomy, the most commonly used technique was laser (90.9%, n = 40), while scissors were used in a limited number of cases (9.1%, n = 4). Lastly, no complications were recorded following the frenotomy in the studied sample. We provided parents with instructions on how to care for and gently massage the spot of the division, following thorough hand hygiene. The recommended frequency for performing these exercises was four to six times per day.

**Table 8:** Absolute and relative frequencies regarding the days of frenotomy, the surgical technique, the type of tongue-tie, and lip-tie.

Descriptive data of frenotomy intervention and classification	n	%
Days until intervention		
1–7 days	16	36.4%
8–14 days	9	20.5%
15–21 days	7	15.9%
22–30 days	10	22.7%
>30 days	2	4.5%
Total	44	100%
Type of tongue-tie		
Type I	7	15.9%
Type II	10	22.7%
Type III	19	43.2%
Type IV	8	18.2%
Total	44	100%
Type of lip-tie		
Type I	-	-
Type II	5	13.2%
Type III	30	78.8%
Type IV	3	7.9%
Total	38	100%
Surgical technique		
Laser	40	90.9%
Scissors	4	9.1%
Total	44	100%

**Figure 1:**
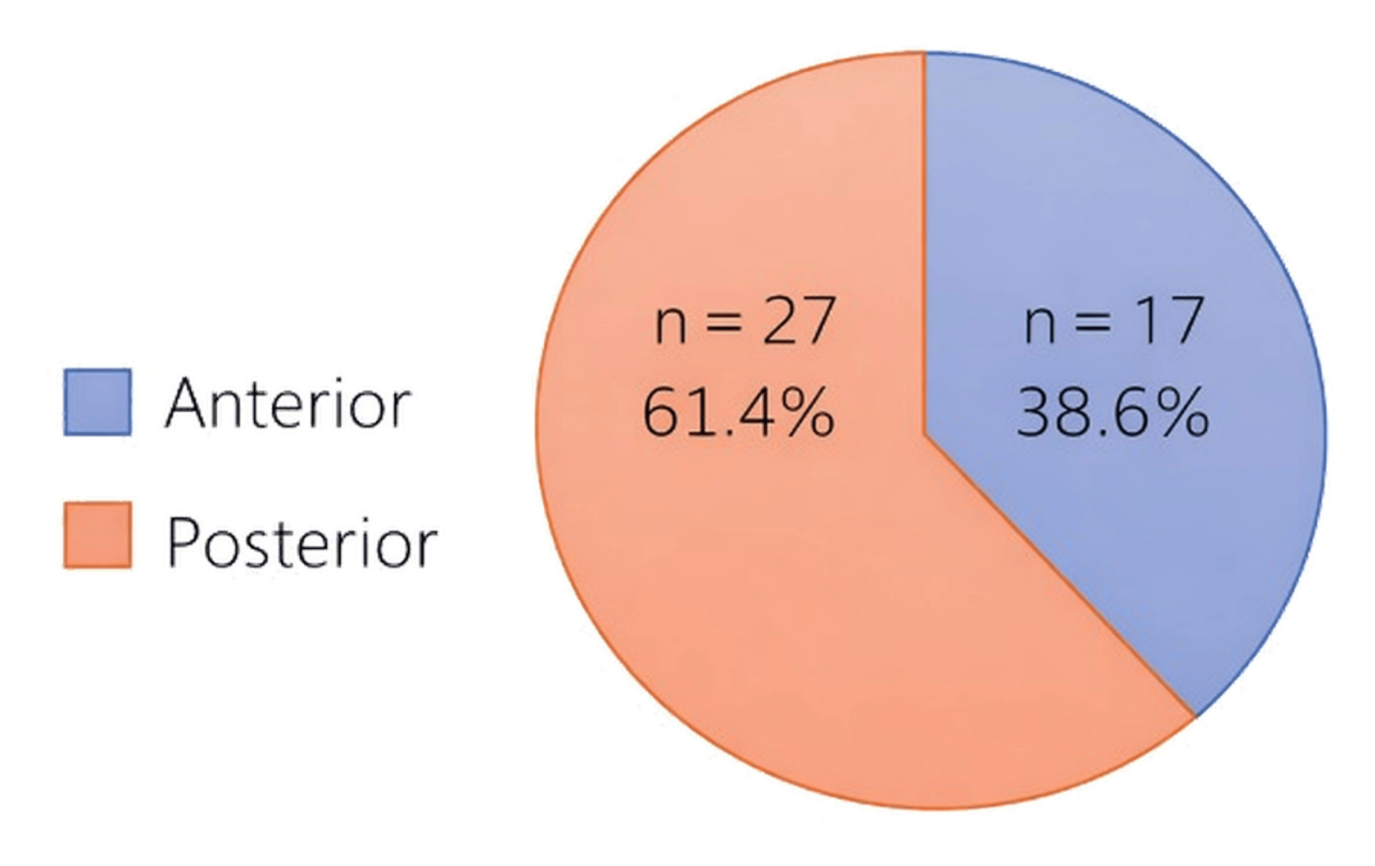
Classification of cases according to the type of ankyloglossia.

In the majority of cases, the frenulum was classified as posterior (61.4%, n = 27), while a smaller proportion was identified as anterior (38.6%, n = 17), based on the Coryllos classification (Figure 1). Regarding the type of lingual frenulum, the most frequently observed type was Type III (43.2%, n = 19), followed by Type II (22.5%, n = 10), Type IV (18.7%, n = 8), and Type I (15.6%, n = 7). As for the type of upper lip frenulum according to Kotlow classification, the dominant type recorded was Type III (78.8%, n = 30), with smaller proportions corresponding to Type II (13.2%, n = 5) and Type IV (7.9%, n = 3) (Table 8).

Breastfeeding symptoms of the mother-infant dyad after the surgical interventions

After the surgical intervention, significant improvement was recorded in many of the issues that had been present as maternal symptoms. Specifically, among the mothers who reported pain throughout breastfeeding before the frenotomy (n = 43), 88.4% (n = 38) experienced complete resolution of the symptom, while 11.6% (n = 5) reported improvement. Similarly, in cases of irritated nipples (n = 48), 97.9% (n = 47) of mothers reported full resolution, and 2.1% (n = 1) noted improvement. Furthermore, among the mothers who experienced cracked or bleeding nipples before the procedure (n = 20), the symptom was completely resolved in all cases (100%, n = 20) (Table 9).

**Table 9:** Absolute and relative frequencies of the maternal symptoms after the surgical intervention. ^1^:Mastitis without an abscess formation.

Maternal symptoms	n	%
Pain during breastfeeding		
Complete resolution	38	88.4%
Partial improvement	5	11.6%
No improvement	-	-
Total	43	100%
Nipple irritation		
Complete resolution	47	97.9%
Partial improvement	1	2.1%
No improvement	-	-
Total	48	100%
Cracked or bleeding nipples		
Complete resolution	20	100%
Partial improvement	-	-
No improvement	-	-
Total	20	100%
Blocked milk ducts		
Complete resolution	4	100%
Partial improvement	-	-
No improvement	-	-
Total	4	100%
Breast engorgement		
Complete resolution	6	85.7%
Partial improvement	1	14.3%
No improvement	-	-
Total	7	100%
Mastitis^1^		
Did not recur	4	100%
Recurred	-	-
Total	4	100%

Following the lingual frenotomy, the progression of symptoms observed in the infants was recorded, as presented in Table 10. Among all infants who had demonstrated inadequate weight gain before the procedure (n = 10), a full recovery (100%) was observed. Similarly, complete resolution was noted in cases of delayed regain of birth weight (n = 9) and weight loss (n = 8). Regarding difficulty in latching onto the breast (n = 42), 95.2% (n = 40) of the infants showed full resolution, while 4.8% (n = 2) showed significant improvement.

**Table 10:** Absolute and relative frequencies of the symptoms concerning the newborn after the surgical intervention. ^1^: Less than 20 g per day. ^2^: Difficulty in regaining birth weight within 14 days after birth. ^3^: More than 10% of the birthweight. ^4^: Prolonged feeding time is defined as more than half an hour in each breast.

Neonatal symptoms	n	%
Ιnadequate weight gain		
Complete resolution	10	100%
Partial improvement	-	-
No improvement	-	-
Total	10	100%
Difficulty regaining birth weight		
Complete resolution	9	100%
Partial improvement	-	-
No improvement	-	-
Total	9	100%
Weight loss^1^		
Complete resolution	8	100%
Partial improvement	-	-
No improvement	-	-
Total	8	100%
Difficulty latching onto the nipple		
Complete resolution	40	95.2%
Partial improvement	2	4.8%
No improvement	-	-
Total	42	100%
Clicking sound during breastfeeding		
Complete resolution	28	96.6%
Partial improvement	1	3.4%
No improvement	-	-
Total	29	100%
Prolonged breastfeeding sessions		
Complete Resolution	40	95.2%
Partial improvement	2	4.8%
No improvement	-	-
Total	42	100%
Supplemental feeding with infant formula		
No longer given	16	84.2%
Reduced	1	5.3%
Continued by the mother’s choice	2	10.5%
Total	19	100%

In cases where a clicking sound of the tongue was present during breastfeeding (n = 29), 96.6% (n = 28) experienced full resolution, and 3.4% (n = 1) reported improvement. Concerning prolonged breastfeeding sessions (n = 42), resolution was observed in 95.2% (n = 40), with improvement noted in 4.8% (n = 2). Finally, with respect to the use of supplemental feeding with formula (n = 19), it was discontinued in 84.2% (n = 16), reduced in 5.3% (n = 1), and continued upon maternal request in 10.5% (n = 2) (Table 10).

Duration of breastfeeding

The overall duration of breastfeeding, the prevalence of exclusive breastfeeding, and the practice of exclusive pumping, categorized by group is presented in Table 11. Group A (mothers and infants who underwent surgical intervention) represents the largest portion of the sample (n = 44). In this group, the majority of mothers (86.4%) breastfed for a duration of more than six months. A total of 70.6% of the participants in this group reported practicing exclusive breastfeeding, suggesting that the intervention appears to have had a positive effect on the maintenance and quality of breastfeeding. Notably, no mother in this group relied exclusively on pumping.

**Table 11:** Absolute and relative frequencies of total breastfeeding duration, categorized by group. ^1^: Group A: Mothers and infants who underwent the intervention involving frenotomy of the lingual and/or upper lip frenulum.^ 2^: Group B: Mothers and infants who declined pediatric surgical evaluation.

Breastfeeding outcomes	Group A^1^		Group B^2^	
	n	%	n	%
Duration of breastfeeding (in months)				
1–3	2	4.5%	2	50%
4–5	4	9.1%	2	50%
6–12	19	43.2%	-	-
13–18	9	20.5%	-	-
19–24	4	9.1%	-	-
25–36	3	6.8%	-	-
≥37	3	6.8%	-	-
Total	44	100%	4	100%
Exclusive breastfeeding				
No	11	25%	4	100%
Yes	33	75%	-	-
Total	44	100%	4	100%
Exclusively pumping				
No	44	100%	2	50%
Yes	-	-	2	50%
Total	44	100%	4	100%

Group B consisted of four cases involving mothers and neonates who did not proceed with pediatric surgical evaluation. In 50% of these cases, breastfeeding was discontinued within the first three months of life, while the remaining 50% ceased breastfeeding between the fourth and fifth month. None of the mothers in this group reported exclusive breastfeeding, and two out of four discontinued direct breastfeeding altogether, opting instead for exclusive pumping (Table 11). These findings may suggest that the absence of intervention potentially had a negative impact on both the duration and quality of breastfeeding, despite the mothers’ initial positive intentions.

Taken together, these findings imply that frenotomy appears to positively influence both the duration and quality of breastfeeding by reducing the reliance on exclusive pumping and increasing the likelihood of successful exclusive breastfeeding.

Correlation between the number of neonatal feeding difficulties and the morphological and functional scores based on the ATLFF assessment tool

A linear regression analysis was conducted to examine the association between the number of neonatal symptoms and the morphological and functional characteristics, as assessed by the ATLFF tool (Table 12). The analysis indicated that the overall model was statistically significant (F = 4.697, df1 = 2, df2 = 47, p = 0.014), explaining 16.7% of the variance in the number of symptoms (R² = 0.167).

**Table 12:** Linear regression analysis of the relationship between the number of neonatal symptoms and ATLFF score. ^1^: R = 0.408, R2 = 0.167, F = 4.697, df1 = 2, df2 = 47, p = 0.014. ^2^: R: Pearson multiple correlation coefficient, R²: Coefficient of determination (explained variance), F: F-statistic, p: Probability of error for regression coefficients. ATLFF = Assessment Tool for Lingual Frenulum Function

ATLFF score	Number of neonatal symptoms				
	B	SE	β	t	P-value
(coefficient α)	3.917	0.803		4.880	<0.001
Μorphological characteristics (ATLFF)	0.311	0.156	0.342	2.002	0.050
Functional characteristics (ATLFF)	-0.345	0.113	-0.522	-3.063	0.004

Regarding the individual predictors, the morphological characteristics assessed by the ATLFF demonstrated a positive trend in relation to the number of symptoms (β = 0.342, t = 2.002, p = 0.050), although this association was not strictly statistically significant at the conventional alpha level (p < 0.05). In contrast, the functional characteristics of the ATLFF showed a negative and statistically significant association with the number of symptoms (β = -0.522, t = -3.063, p = 0.004), suggesting that better functional performance of the lingual frenulum is associated with fewer neonatal or infant symptoms.

These findings imply that the morphological features of the lingual frenulum, as evaluated through the ATLFF tool, are related to the number of symptoms observed in the neonate, i.e., the more abnormal the morphological features, the greater the number of symptoms tends to be. However, this relationship is not statistically robust. On the other hand, the functional characteristics of the frenulum presented a clear and statistically significant negative correlation with the presence of symptoms. In other words, better functional mobility of the frenulum is associated with fewer breastfeeding-related difficulties, indicating that the functional assessment may be a more critical determinant in understanding neonatal feeding issues.

Correlation between the number of maternal difficulties and the morphological and functional scores based on the ATLFF assessment tool

By using linear regression analysis, we examine the relationship between the number of maternal symptoms and the morphological and functional characteristics, as assessed using the ATLFF tool (Table 13). The analysis indicated that the overall model was marginally statistically significant (F = 2.507, df1 = 2, df2 = 48, p = 0.050) and explained 9.5% of the variance in the number of maternal symptoms (R² = 0.095). The morphological characteristics assessed by the ATLFF showed a positive trend in relation to the number of maternal symptoms (β = 0.335, t = 1.911, p = 0.062), although this finding approached but did not meet conventional significance criteria (p < 0.05). In contrast, the functional characteristics of the ATLFF demonstrated a negative and statistically significant association with the number of maternal symptoms (β = -0.368, t = -2.101, p = 0.041), suggesting that better functional performance of the lingual frenulum is associated with fewer breastfeeding-related difficulties experienced by the mother.

**Table 13:** Linear regression analysis of the relationship between the number of maternal symptoms and ATLFF score. ^1^: R = 0.308, R2 = 0.095, F = 2.507, df1 = 2, df2 = 48, p = 0.050. ^2^: R: Pearson multiple correlation coefficient, R²: Coefficient of determination (explained variance), F: F-statistic, p: Probability of error for regression coefficients. ATLFF = Assessment Tool for Lingual Frenulum Function

ATLFF score	Number of maternal symptoms				
	b	SE	β	t	P-value
(coefficient α)	2.534	0.511		4.961	<0.001
Μorphological characteristics	0.187	0.098	0.335	1.911	0.062
Functional characteristics	-0.150	0.078	-0.368	-2.101	0.041

This indicates that the statistical model used to interpret the factors influencing the symptoms reported by the mother is marginally statistically significant. In other words, its results are not particularly strong but reveal a noteworthy trend. The model explains approximately 9.5% of the total variance in the number of symptoms reported by the mother, a relatively small but non-negligible proportion. The functional characteristics of the lingual frenulum exhibited a negative and statistically significant association with maternal symptoms. In other words, the better the functional performance of the lingual frenulum, the fewer difficulties are reported by the mother, and this relationship is statistically reliable (p = 0.041). This suggests that functional characteristics are more strongly associated with maternal difficulties than morphological characteristics.

## Discussion

The present findings emphasize the importance of oral cavity examination by trained midwives, detailed breastfeeding history, and direct observation of the breastfeeding dyad in order to discern which infants will benefit from frenotomy. The significant associations found between functional tongue restriction and both maternal and neonatal symptoms support the inclusion of functional screening tools such as ATLFF and the newly developed TOMARA questionnaire in clinical practice. Midwives, as frontline providers of breastfeeding support, are uniquely positioned to recognize signs of impaired tongue mobility and to refer cases appropriately, thereby preventing delayed diagnoses and promoting breastfeeding success.

The diagnosis and management of ankyloglossia appear to be quite complex. However, if the lingual frenulum is categorized into two main types, anterior and posterior, instead of four, the associated challenges may become more clearly discernible. The diagnosis of anterior types of ankyloglossia tends to be more straightforward, as the condition is visually apparent and presents with a distinct deviation from typical anatomical structures [15]. The characteristic heart-shaped appearance of the tongue or the presence of a central notch, along with a frenulum attached near or at the tip of the tongue, has been closely associated with neonatal cases of ankyloglossia [7,16]. Conversely, when the frenulum is classified as posterior, the anatomical structures tend to resemble those of a normal presentation, rendering referral decisions more challenging and resulting in such cases being less frequently identified. In these cases, the tongue appears rounded, and the frenulum is attached toward the midline of the tongue, a configuration often perceived as anatomically typical, without any evident abnormalities [17]. For some healthcare professionals, these morphological observations may suffice to classify a newborn as not being tongue-tied. However, are such anatomical findings sufficient? In identifying cases of ankyloglossia, it is imperative to incorporate data on tongue functionality and breastfeeding difficulties.

The association between ankyloglossia and breastfeeding difficulties has been studied extensively. Ankyloglossia, which may contribute to breastfeeding challenges, is described in the literature as either symptomatic or asymptomatic. The term “symptomatic” refers to cases where tongue mobility is significantly restricted by the frenulum, resulting in persistent difficulties that cannot be resolved through individualized lactation counseling alone [18]. A clearer distinction between a true case of neonatal ankyloglossia and general breastfeeding challenges is provided by the limited mobility of the tongue, specifically, its restricted upward, forward, and lateral movement, reflecting the range of motion permitted by the frenulum [19]. Findings from the present study suggest that functional characteristics of the tongue, rather than morphological features alone, are more helpful in differentiating cases of ankyloglossia that require intervention. Emphasis on tongue functionality over isolated anatomical traits has also been advocated by Mills [1]. In neonates with ankyloglossia who breastfeed effectively without notable difficulties, no therapeutic intervention is required. However, in symptomatic cases where lactation counseling or orofacial myofunctional therapy does not yield improvements, frenotomy is recommended. This decision should be made following informed parental consent, as emphasized by the Academy of Breastfeeding Medicine [9].

In cases where tongue mobility is borderline, the binary dilemma of whether to cut or not to cut is supplemented by the option of continued monitoring, in collaboration with the mother, throughout the breastfeeding process. If the newborn with marginal functional limitations is able to breastfeed effectively and difficulties remain mild, intervention is not necessary [18]. The duration of this monitoring period should be tailored based on the number and severity of the dyad’s challenges. As demonstrated in studies involving control groups, many infants with tongue-tie are able to breastfeed effectively without requiring surgical intervention [20-22].

Riskin and colleagues concluded that neonates with ankyloglossia, regardless of the frenulum’s attachment point on the tongue, are more likely to encounter breastfeeding challenges within the first 30 days of life [23]. Ankyloglossia is not responsible for every breastfeeding difficulty encountered by a mother-infant dyad [2]. Very often, differential diagnosis reveals that breastfeeding challenges initially attributed to ankyloglossia are actually the result of poor latch and positioning, flat or inverted nipples, and milk supply issues. In such cases, individualized breastfeeding counseling is usually sufficient to resolve the issue. Due to heightened awareness surrounding ankyloglossia, many breastfeeding mothers now request an evaluation of their newborn’s frenulum before seeking lactation support. Conversely, some mothers who repeatedly seek help for persistent breastfeeding difficulties and are merely advised to use nipple cream and be patient, often without a thorough assessment or adequate support.

The continuation of breastfeeding appears to be associated with frenotomy and a reduction in nipple pain, considering that nipple pain is one of the primary reasons for early cessation of breastfeeding [24,25]. This conclusion is supported by a large Cochrane review as well as several smaller studies reporting statistically significant outcomes [20,24,26,27]. However, in a large retrospective study involving 2,333 neonates, frenotomy was not associated with prolonged breastfeeding, but rather with non-exclusive breastfeeding [28]. In the present study, frenotomy was associated with both a longer breastfeeding duration beyond six months (86.4%) and higher rates of exclusive breastfeeding (70.6%). In the current literature, this association remains unclear, largely due to the limited number of randomized controlled trials available.

In Greece, frenotomy is performed by pediatric surgeons and otolaryngologists, whereas in other countries, a broader range of healthcare professionals are involved in this procedure [29]. In the present study, frenotomy was well tolerated by all participating neonates, with no reported complications. The procedure resulted in improvement of all symptoms, and in the majority of cases, it led to complete resolution. We believe that this favorable outcome is largely attributable to the careful selection of the study sample.

Limitations of the study

One of the main limitations of this study is the relatively small sample size (N = 51), which reduced the statistical power for conducting multivariable regression analyses and for adjusting for potential confounding factors, including prior breastfeeding experience, maternal education, and gestational age. Consequently, the statistical associations reported should therefore be interpreted as exploratory findings rather than causal relationships. The small sample size is primarily due to the strict inclusion and exclusion criteria of the study, the requirement for continuous postpartum follow-up, and the fact that ankyloglossia, while clinically significant, affects a relatively small proportion of the neonatal population. Recruiting a larger, more homogeneous sample under similar methodological conditions is both time-consuming and resource-intensive.

Furthermore, the limitations of our study are consistent with those commonly observed in single-group designs. Specifically, the absence of a large control group and the lack of randomization represent significant methodological challenges. Given the ongoing debate surrounding ankyloglossia and the frequent mention in systematic reviews of the lack of studies that thoroughly examine the characteristics of affected neonates, we chose to focus on a specific sample of infants. This was achieved through a focused evaluation of maternal and neonatal outcomes both before and after surgical intervention, with particular attention to exclusive and non-exclusive breastfeeding duration. Future research with larger and more diverse cohorts is recommended to validate these findings and allow for more advanced statistical modeling, including control for potential confounders. Such efforts will be essential for establishing evidence-based guidelines for the diagnosis and management of ankyloglossia in the neonatal period.

## Conclusions

Midwives can play an active role in identifying neonates with ankyloglossia and referring them to the appropriate healthcare professionals. At the same time, collaboration with pediatricians, pediatric surgeons, and otolaryngologists is essential, thereby forming a multidisciplinary and individualized approach to neonatal ankyloglossia. Ongoing education in breastfeeding support and counseling is fundamental for effective practice. Continuous observation of breastfeeding, combined with systematic oral cavity examinations, enables professionals to gain experience in recognizing anatomical and functional variations of the oral structures and lingual frenulum. The better the functional performance of the lingual frenulum, the fewer difficulties are recorded by the mother and the infant. The core dilemma is not merely whether to perform a frenotomy, but whether to perform it, to defer it, or to ensure appropriate follow-up of the breastfeeding dyad.

## Figures and Tables

**Figure 2:**
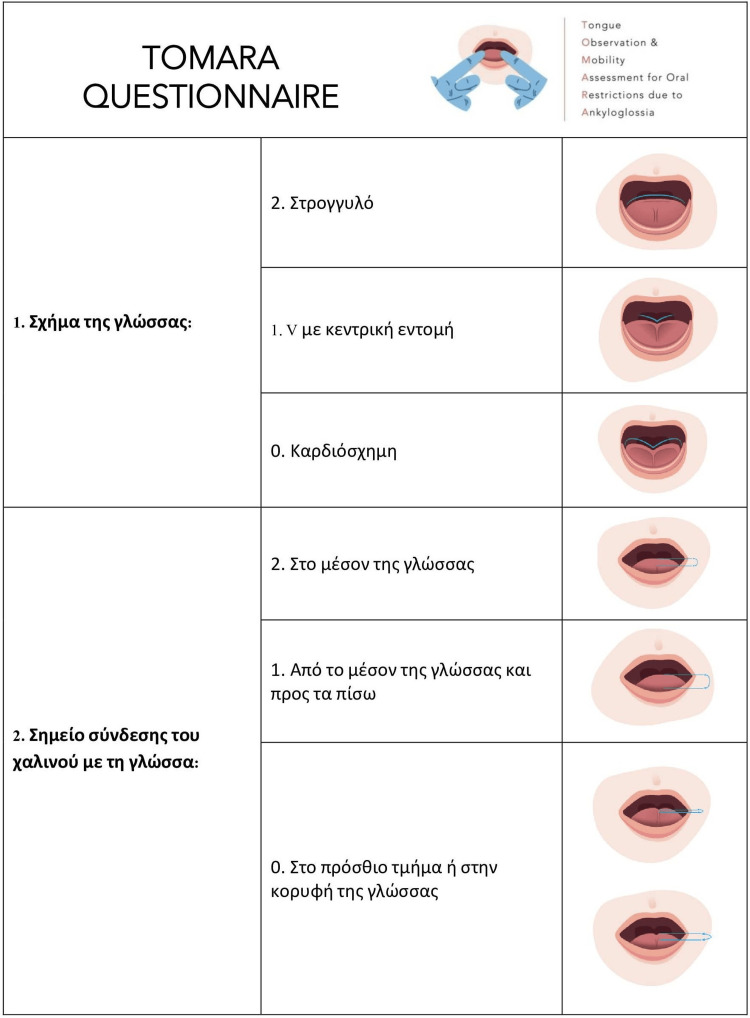
TOMARA questionnaire (Greek version, items 1 and 2).

**Figure 3:**
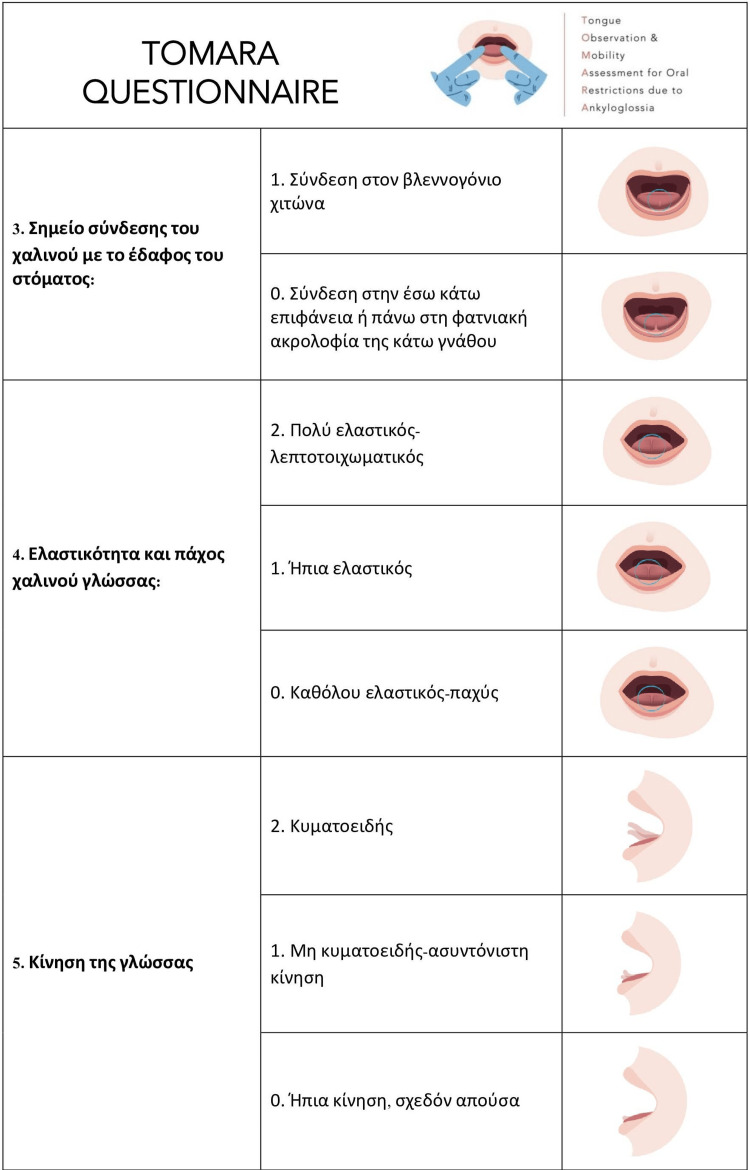
TOMARA questionnaire (Greek version, items 3–5).

**Figure 4:**
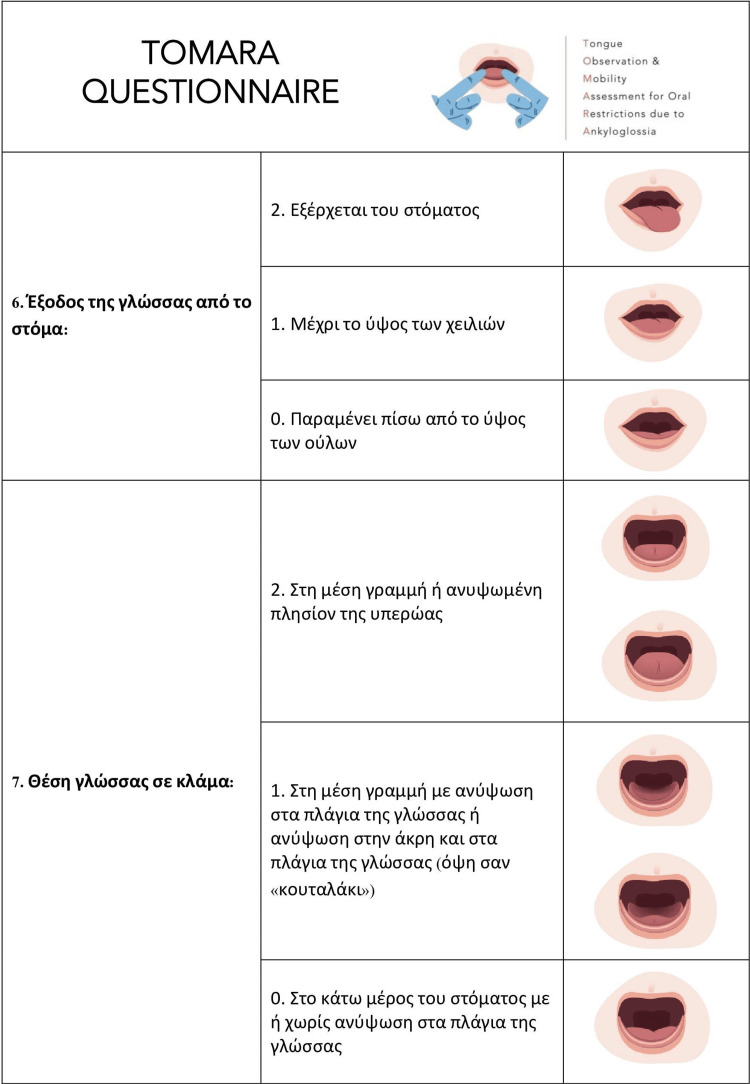
TOMARA questionnaire (Greek version, items 6 and 7).

**Figure 5:**
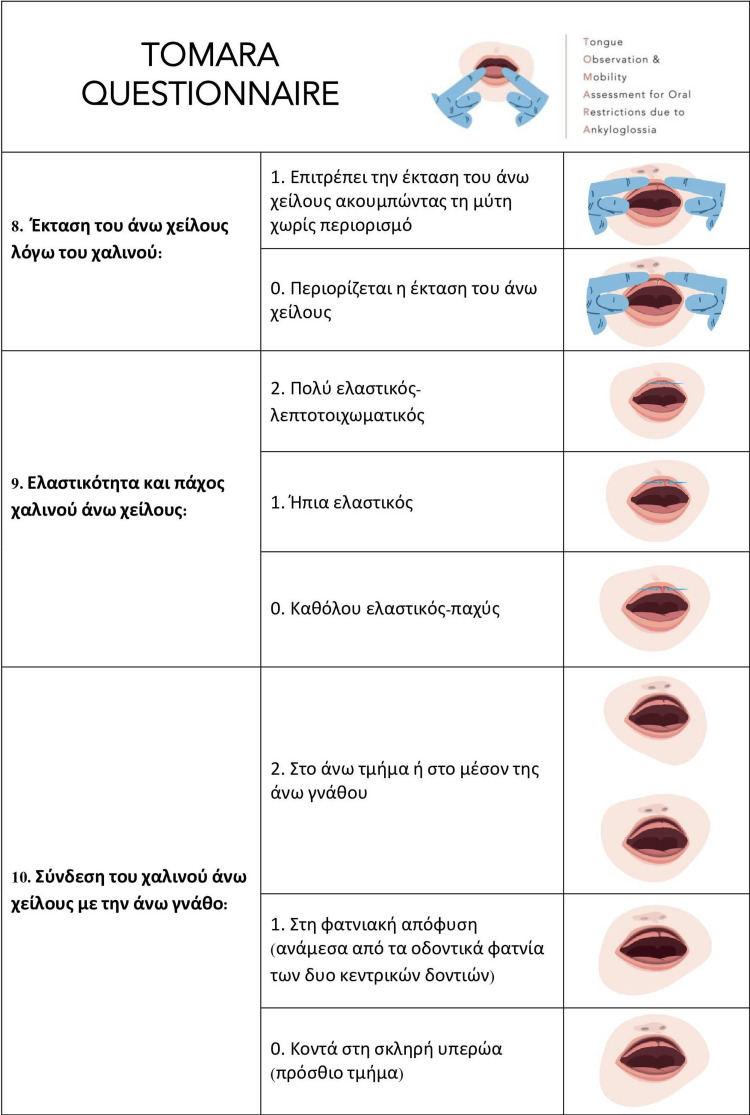
TOMARA questionnaire (Greek version, items 8–10).

**Figure 6:**
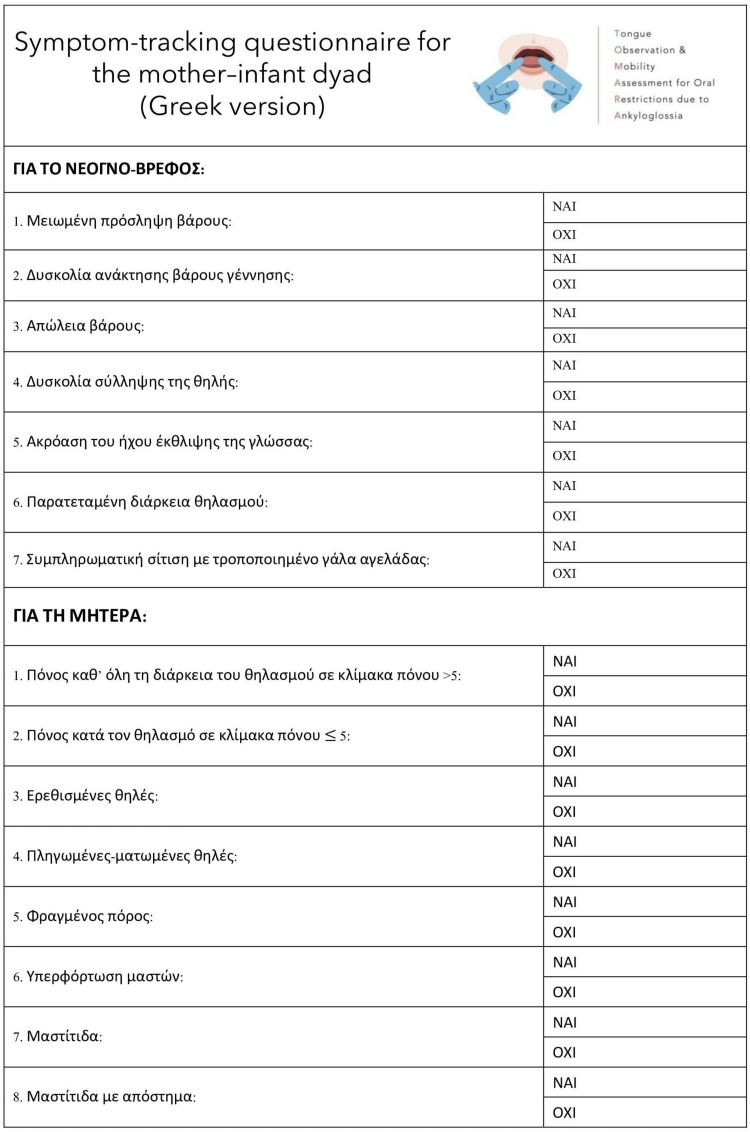
Structured symptom-tracking questionnaire for the mother–infant dyad prior to frenotomy (Greek version).

**Figure 7:**
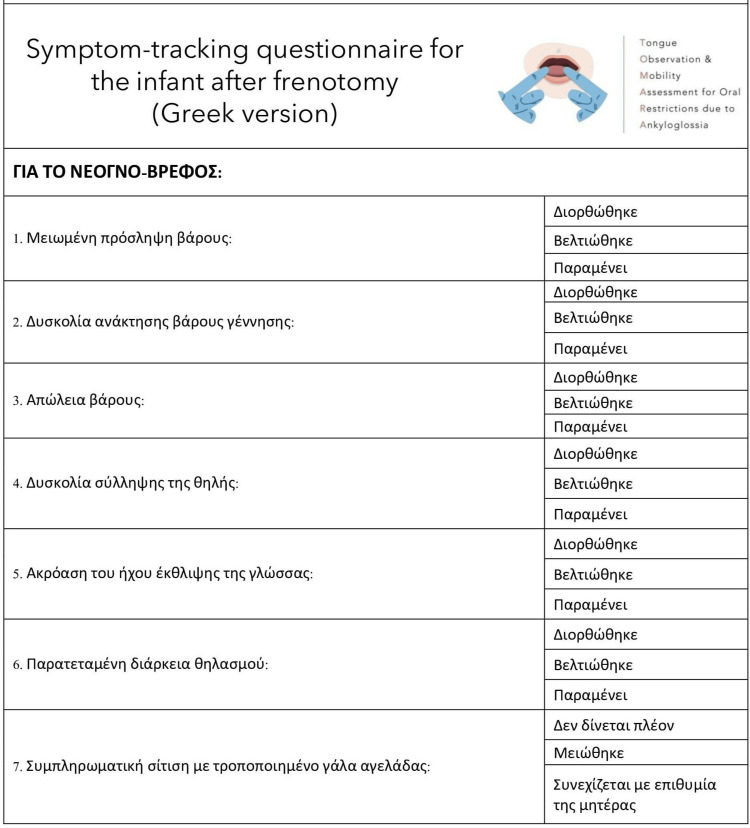
Structured symptom-tracking questionnaire for the infant following frenotomy (Greek version).

**Figure 8:**
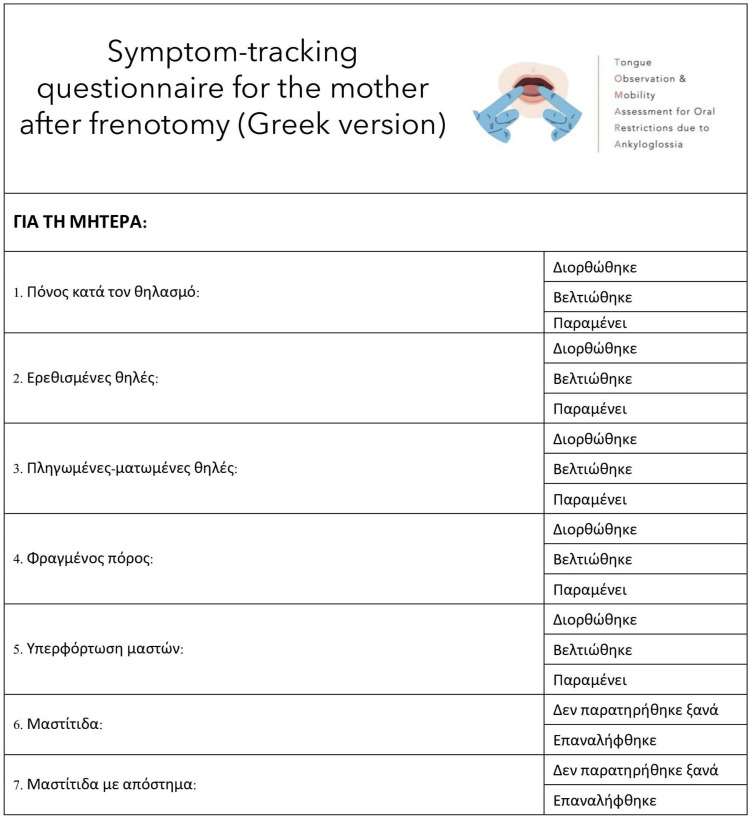
Structured symptom-tracking questionnaire for the mother following frenotomy (Greek version).

## Appendices

 Appendix 1

Appendix 2 

Appendix 3 

Appendix 4 

Appendix 5 

Appendix 6 

Appendix 7 

## References

[1] Mills N, Pransky SM, Geddes DT, Mirjalili SA (2019). What is a tongue tie? Defining the anatomy of the in-situ lingual frenulum. Clin Anat.

[2] Messner AH, Walsh J, Rosenfeld RM (2020). Clinical consensus statement: ankyloglossia in children. Otolaryngol Head Neck Surg.

[3] Becker S, Brizuela M, Mendez MD (2023). Ankyloglossia (Tongue-Tie). https://www.ncbi.nlm.nih.gov/books/NBK482295/.

[4] (2025). World Health Organization. Breastfeeding. https://www.who.int/health-topics/breastfeeding.

[5] Christian P, Smith ER, Lee SE, Vargas AJ, Bremer AA, Raiten DJ (2021). The need to study human milk as a biological system. Am J Clin Nutr.

[6] Witkowska-Zimny M, Kaminska-El-Hassan E (2017). Cells of human breast milk. Cell Mol Biol Lett.

[7] Brzęcka D, Garbacz M, Micał M, Zych B, Lewandowski B (2019). Diagnosis, classification and management of ankyloglossia including its influence on breastfeeding. Dev Period Med.

[8] Talmor G, Caloway CL (2022). Ankyloglossia and tethered oral tissue: an evidence-based review. Pediatr Clin North Am.

[9] LeFort Y, Evans A, Livingstone V (2021). Academy of Breastfeeding Medicine Position Statement on Ankyloglossia in Breastfeeding Dyads. Breastfeed Med.

[10] Walsh J, Tunkel D (2017). Diagnosis and treatment of ankyloglossia in newborns and infants: a review. JAMA Otolaryngol Head Neck Surg.

[11] Hazelbaker AK (1993). The Assessment Tool for Lingual Frenulum Function (ATLFF): Use in a Lactation Consultant Private Practice. The assessment tool for lingual frenulum function (ATLFF): Use in a lactation consultant private practice [thesis].

[12] MacCallum RC, Widaman KF, Zhang S, Hong S (1999). Sample size in factor analysis. Psych Meth.

[13] Gorsuch RL (1997). Exploratory factor analysis: its role in item analysis. J Pers Assess.

[14] Tabachnick BG, Fidell LS (2001). Using Multivariate Statistics, 4th Wd. Using Multivariate Statistics, 4th ed.; Allyn and Bacon: Needham Heights.

[15] Hong P, Lago D, Seargeant J, Pellman L, Magit AE, Pransky SM (2010). Defining ankyloglossia: a case series of anterior and posterior tongue ties. Int J Pediatr Otorhinolaryngol.

[16] Martinelli RL, Marchesan IQ, Berretin-Felix G (2020). Tongue position for lingual frenulum assessment. Rev CEFAC.

[17] Coryllos EV, Genna CW, Fram JL (2016). Minimally invasive treatment for posterior tongue-tie (the hidden tongue-tie). Supporting Sucking Skills in Breastfeeding Infants.

[18] Thomas J, Bunik M, Holmes A, Keels MA, Poindexter B, Meyer A, Gilliland A (2024). Identification and management of ankyloglossia and its effect on breastfeeding in infants: clinical report. Pediatrics.

[19] Hentschel R (2018). Breastfeeding problems should be the only relevant criteria for deciding whether to carry out a frenotomy in infancy. Acta Paediatr.

[20] Buryk M, Bloom D, Shope T (2011). Efficacy of neonatal release of ankyloglossia: a randomized trial. Pediatrics.

[21] Emond A, Ingram J, Johnson D, Blair P, Whitelaw A, Copeland M, Sutcliffe A (2014). Randomised controlled trial of early frenotomy in breastfed infants with mild-moderate tongue-tie. Arch Dis Child Fetal Neonatal Ed.

[22] Hogan M, Westcott C, Griffiths M (2005). Randomized, controlled trial of division of tongue-tie in infants with feeding problems. J Paediatr Child Health.

[23] Riskin A, Mansovsky M, Coler-Botzer T (2014). Tongue-tie and breastfeeding in newborns-mothers' perspective. Breastfeed Med.

[24] O'Shea JE, Foster JP, O'Donnell CP, Breathnach D, Jacobs SE, Todd DA, Davis PG (2017). Frenotomy for tongue-tie in newborn infants. Cochrane Database Syst Rev.

[25] Odom EC, Li R, Scanlon KS, Perrine CG, Grummer-Strawn L (2013). Reasons for earlier than desired cessation of breastfeeding. Pediatrics.

[26] Ghaheri BA, Cole M, Fausel SC, Chuop M, Mace JC (2017). Breastfeeding improvement following tongue-tie and lip-tie release: a prospective cohort study. Laryngoscope.

[27] O'Callahan C, Macary S, Clemente S (2013). The effects of office-based frenotomy for anterior and posterior ankyloglossia on breastfeeding. Int J Pediatr Otorhinolaryngol.

[28] Guinot F, Carranza N, Ferrés-Amat E, Carranza M, Veloso A (2022). Tongue-tie: incidence and outcomes in breastfeeding after lingual frenotomy in 2333 newborns. J Clin Pediatr Dent.

[29] Hale M, Mills N, Edmonds L, Dawes P, Dickson N, Barker D, Wheeler BJ (2020). Complications following frenotomy for ankyloglossia: a 24-month prospective New Zealand Paediatric Surveillance Unit study. J Paediatr Child Health.

